# Hand Control With Invasive Feedback Is Not Impaired by Increased Cognitive Load

**DOI:** 10.3389/fbioe.2020.00287

**Published:** 2020-04-03

**Authors:** Giacomo Valle, Edoardo D’Anna, Ivo Strauss, Francesco Clemente, Giuseppe Granata, Riccardo Di Iorio, Marco Controzzi, Thomas Stieglitz, Paolo M. Rossini, Francesco M. Petrini, Silvestro Micera

**Affiliations:** ^1^Bertarelli Foundation Chair in Translational Neuroengineering, Center for Neuroprosthetics and Institute of Bioengineering, School of Engineering, École Polytechnique Fédérale de Lausanne (EPFL), Lausanne, Switzerland; ^2^The BioRobotics Institute and Department of Excellence in AI and Robotics, Scuola Superiore Sant’Anna, Pisa, Italy; ^3^Laboratory for Neuroengineering, Department of Health Sciences and Technology, Institute for Robotics and Intelligent Systems, ETH Zürich, Zurich, Switzerland; ^4^Department of Excellence in Robotics and AI, Scuola Superiore Sant’Anna, Pisa, Italy; ^5^Institute of Neurology, Catholic University of The Sacred Heart, Policlinic A. Gemelli Foundation, Rome, Italy; ^6^Laboratory for Biomedical Microtechnology, Department of Microsystems Engineering–IMTEK, Bernstein Center, BrainLinks-BrainTools Cluster of Excellence, University of Freiburg, Freiburg im Breisgau, Germany; ^7^Area of Neuroscience, IRCCS San Raffaele Pisana, Rome, Italy

**Keywords:** neural sensory feedback, superficial sensory feedback, upper limb amputees, prosthesis, cognitive load, neural interfaces, electrical stimulation

## Abstract

Recent experiments have shown that neural stimulation can successfully restore sensory feedback in upper-limb amputees improving their ability to control the prosthesis. However, the potential advantages of invasive sensory feedback with respect to non-invasive solutions have not been yet identified. Our hypothesis was that a difference would appear when the subject cannot focus all the attention to the use of the prosthesis, but some additional activities require his/her cognitive attention, which is a quite common situation in real-life conditions. To verify this hypothesis, we asked a trans-radial amputee, equipped with a bidirectional hand prosthesis, to perform motor tasks also in combination with a cognitive task. Sensory feedback was provided via intraneural (invasive) or electro-tactile (non-invasive) stimulation. We collected also data related to self-confidence. While both approaches were able to significantly improve the motor performance of the subject when no additional cognitive effort was asked, the manual accuracy was not affected by the cognitive task only when intraneural feedback was provided. The highest self-confidence was obtained when intraneural sensory feedback was provided. Our findings show that intraneural sensory feedback is more robust to dual tasks than non-invasive feedback. This is the first direct comparison between invasive and non-invasive approaches for restoring sensory feedback and it could suggest an advantage of using invasive solutions.

**Clinical Trial Registration:** www.ClinicalTrials.gov, identifier NCT02848846.

## Introduction

The loss of a hand affects persons’ quality of life (Meyer, 2003). Several clinical solutions have been provided compared to the first manufactured prostheses, developing more dexterous artificial hands (Belter et al., 2013). Nevertheless, the lack of sensory information flow from the missing hand is still among the reasons for prosthesis underuse (Biddiss and Chau, 2007). To this aim, several invasive (Raspopovic et al., 2014; Tan et al., 2014; Davis et al., 2016; Petrini et al., 2018) and non-invasive (Marasco et al., 2018; Osborn et al., 2018) technologies have been developed to restore sensory feedback in upper limb amputees. Different implantable peripheral interfaces have been shown to efficiently stimulate the sensory nerves restoring natural sensations (Raspopovic et al., 2014; Tan et al., 2014; Valle et al., 2018) optimally integrated (Risso et al., 2019), improving prosthesis control (Tan et al., 2014; Petrini et al., 2018; Valle et al., 2018) and prosthesis embodiment (Valle et al., 2018), diminishing phantom limb pain (Petrini et al., 2018) also in chronic, long-lasting applications (Tan et al., 2014; Petrini et al., 2018).

**FIGURE 1:**
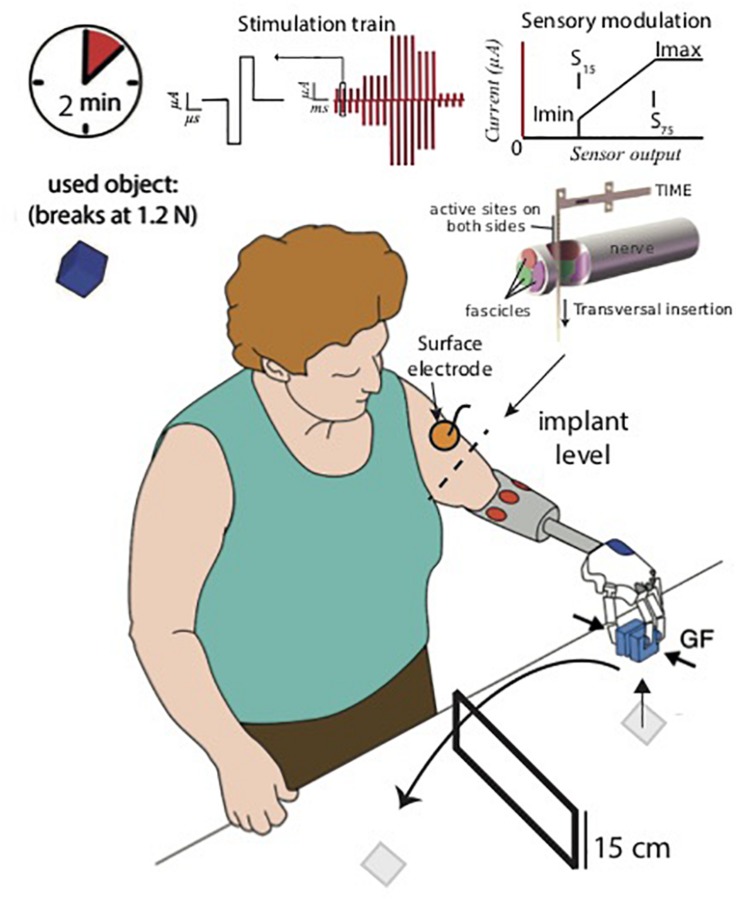
Bidirectional hand prosthesis. During the tests, the patient used a robotic hand prosthesis controlled through surface EMG signals, and providing neural sensory feedback through a single channel of a TIME implanted in her ulnar nerve or electrotactile sensory feedback through a single skin-electrode placed on her residual arm. The subject performed a dual-task paradigm involving motor and memory skills simultaneously (VET + SDFT, respectively).

However, since daily activities are frequently performed in a “dual-task” paradigm condition (i.e., holding a beer while reading a book) (Land et al., 1999), the execution of motor tasks with a bidirectional prosthesis should be assessed in combination with tasks increasing the cognitive load for the user. To accomplish this dual paradigm the user cannot (or should not) focus all the attention to the use of the prosthesis. This is a very important issue but, so far, the impact of cognitive efforts on the efficacy of the ongoing artificial sensory feedback has not been investigated. Here, we evaluate the effect of using a bidirectional hand prosthesis with intraneural, with non-invasive electrotactile or with no sensory feedback in a dual-task paradigm in which subjects can use the visual feedback (Figure 1). A trans-radial amputee was recruited and implanted with transversal intrafascicular multichannel electrodes [TIME (Petrini et al., 2018; Valle et al., 2018)]. In particular, we assessed the short-term memory capacity [STC or *memory span* (Lovett et al., 2000; Jones and Macken, 2015)] while the patient was performing a motor task [Virtual Eggs Test (Clemente et al., 2016)] requiring manual accuracy and dexterity in three different sensory feedback conditions (Figure 1). We provided to the amputee: (i) intraneural sensory feedback (IF) delivering stimulation trains through an implanted TIME in the ulnar nerve, or (ii) electrotactile sensory feedback (SF) delivering electrical stimulation through a surface electrode placed on the residual arm skin or (iii) no sensory feedback (NF). We measured patient’s performance reporting memory span, manual accuracy and manual dexterity achieved during the dual-task. Results indicate that only intraneural sensory feedback could allow to achieve a robust improvement in motor performance, maintaining a high STC, also in case of increased cognitive load.

## Methods

### Patient Recruitment and Surgical Procedures

A single patient participated in this study after providing her informed consent: a right-handed 54-year-old female with a distal two-thirds of the left forearm trans-radial amputation incurred 2 years prior to the study.

Briefly, the subject was implanted with four TIMEs, two in the median nerve and two in the ulnar nerve (above the elbow). Overall, 56 active sites were available (14 per electrode). They were implanted on June 24th 2017. The explant of the electrodes was executed on December 16th 2017. The electrode cable segments were located in subcutaneous pockets, externalized (and secured with sutures and subcutaneous strain release loops) in order to be available for the transcutaneous connection with a neural stimulator (Petrini et al., 2018) (Ripple LLC).

### Prosthesis Movement Control

The bidirectional control setup is described in detail in Valle et al. (2018). Briefly, for prosthesis control, surface electromyographic signals (sEMG) were acquired from the forearm muscles and decoded using a k-NN classifier (3 classes: close, open and rest). The control algorithm was the same in each condition. The decoded movement was sent to a prosthetic hand (IH2 Azzurra, Prensilia, Italy), equipped with tension and position sensors in each digit. Based on the recorded position and tension information, stimulation pulses were delivered through the four TIME electrodes.

### Neural and Electrotactile Sensory Feedback

**FIGURE 2:**
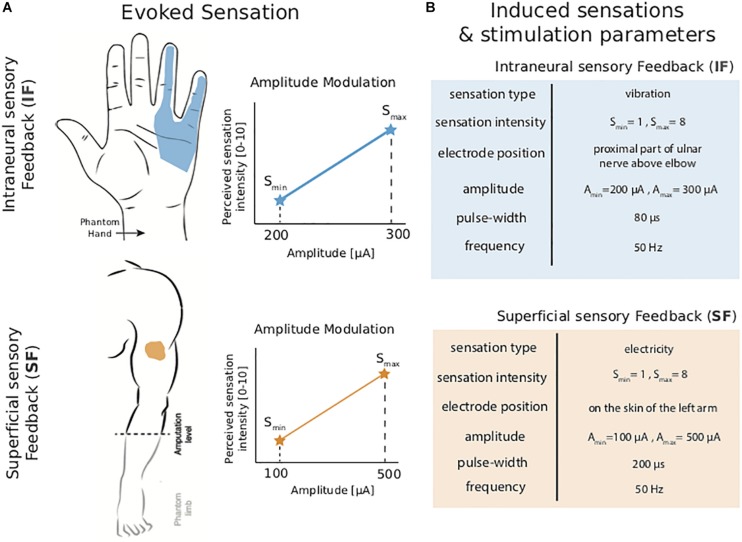
Sensory feedback. **(A)** Locations of the elicited sensation when Intraneural sensory Feedback (IF) and Superficial sensory Feedback were provided. Both encoding strategies exploited amplitude modulation. **(B)** Induced sensation and stimulation parameters were reported for intraneural stimulation (IF) and superficial electrotactile stimulation (SF).

Intraneural tactile feedback was delivered using the same setup described inValle et al. (2018). After an extensive mapping phase, as described in Petrini et al. (2018) andValle et al. (2018) we identified the optimal stimulation parameters for each channel, such as perceptual threshold stimulation charge and pain thresholds. Stimulation frequency was always fixed at 50 Hz as in previous studies (Raspopovic et al., 2014), and injected current levels were always below the chemical safety limit of 120 nC (Petrini et al., 2018). To achieve sensation modulation, the injected charge was modulated by changing the injected current amplitude. Rectangular, biphasic stimulation pulse amplitudes were modulated between 200 and 300 μA with a fixed pulse width of 80 μs. In all cases, the resulting sensation was described as pressure or vibration referred to most of the ulnar innervation area (Figure 2A). The patient reported a direct proportional correlation between the amplitude of the stimulation injected and the intensity of the evoked sensation, producing a very limited increase of the extent of the area of the elicited sensations [details reported in Petrini et al. (2018)].

For a non-invasive approach an electrotactile stimulation was used placing a surface electrode on the arm. Stimulation was delivered as square charge balanced biphasic pulse trains with a fixed frequency [50 Hz as in D’Anna et al. (2017)], in such a way as to elicit only *in loco* sensation under the electrode. Perceptual thresholds and pain thresholds were identified using the same approach used for intraneural stimulation and described in Petrini et al. (2018). The amplitude used varied between 100 μA and 500 μA, with a pulse width duration of 200 μs (Figure 2A).

Before these experiments the patient already exploited both IF and SF in a bidirectional hand prosthesis during extensive tests (Petrini et al., 2018; D’Anna et al., 2019).

### Dual-Task Paradigm

In the dual-task paradigm, the patient was asked to perform the Virtual Eggs Test(VET) and the Span Digit Forward Test [SDFT (Blackburn and Benton, 1957)] simultaneously. The VET is a recently proposed test for sensorimotor assessment (Clemente et al., 2016). During the VET, the patient, wearing the prosthesis, was instructed to transfer the fragile blocks presented in front of her from one side to the other over a 15 cm tall wall as fast as possible and without breaking them (Figure 1). The performances were measured by the number of transferred (broken and unbroken) blocks (gross manual dexterity, D) and the number of transferred unbroken blocks over the total number of blocks (manual accuracy, A) during 2-min trials. In this work, the virtual eggs would break when grasped with a grip force larger than 1.2 N, which was determined in Petrini et al. (2018). This motor task was previously presented and used to assess motor performance in transradial amputees (Petrini et al., 2018; Valle et al., 2018).

**FIGURE 3:**
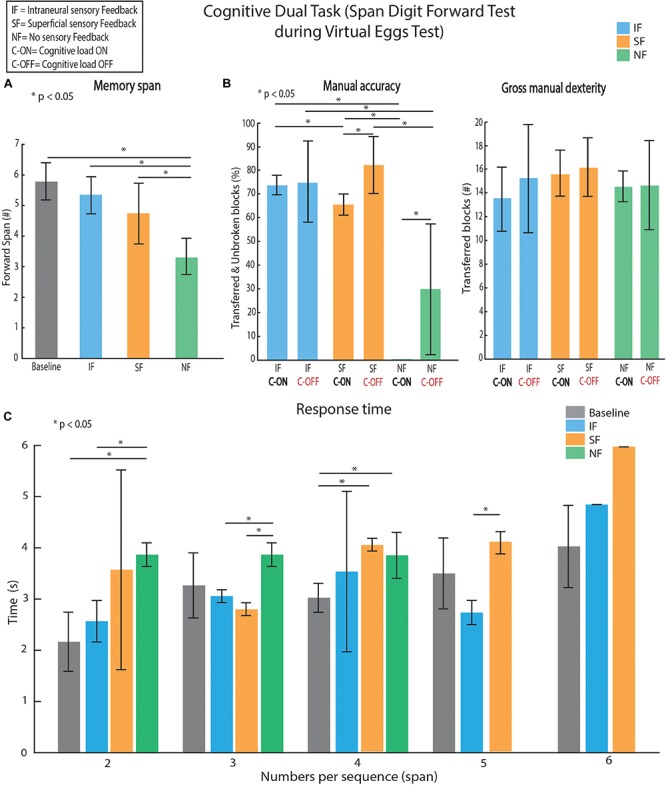
Dual-task: motor control and short-term memory assessment. **(A)** Memory digit spans according to the different conditions are presented. Baseline was acquired as control value. **(B)** Performances are evaluated as manual accuracy (number of unbroken and transferred blocks over total transferred blocks) and manual dexterity (number of total transferred blocks). **(C)** Time to recall all the digits sequence was collected in each condition. All data are reported as mean values ± standard deviations. A span of 6 was reached only once in SF and once in IF (error bars without std). Friedman test, with Tukey-Kramer correction for multiple groups of data when requested, was performed. We performed 5 repetitions × 15 feedback conditions (Intraneural Feedback – IF, Superficial Feedback – SF and No Feedback – NF) × 2 cognitive conditions (with Cognitive task C-ON and without Cognitive task C-OFF).

During the VET, the patient was asked to repeat digits in the order in which they were read out by the experimenter (SDFT). The digits were asked in an even tone, at approximately the rate of one digit per second. The patient’s memory span was defined as the maximum length of the digit lists of which the patient recalled correctly (Lezak et al., 2012). The memory span at rest condition was also reported as a baseline. The response time, defined as the time requested by the patient to repeat the entire digits list, was collected in each condition using a chronometer. The patient performed 15 trials for each condition (IF, SF, and NF). Each condition was tested in a different day by the patient after several tests for each of them (Petrini et al., 2018; D’Anna et al., 2019). The VET performances were evaluated in each stimulation condition with (C-ON) and without (C-OFF) SDFT (Figure 3B).

For each trial set, after the task we asked the subject to rate her confidence in her ability to perform the task, on a scale of 0 to 10 (Schiefer et al., 2018).

### Statistical Analysis

All data were analyzed using MATLAB (R2016a, The MathWorks, Natick, MA, United States). All statistics were performed using the available built-in functions. The data were not normally distributed, the *p*-values of the Kolmogorov–Smirnov test were always >0.1. Thus, the Friedman test, with Tukey-Kramer correction for multiple groups of data when requested, was performed. All reported *p*-values resulting from the Friedman test (p) measure the significance of the statistic. The number of repetitions for each experiment is reported in the corresponding figure captions.

### Data Availability Statement

The datasets (also protocol and statistical analysis plan) generated during and/or analyzed during the current study are available from the corresponding author on reasonable request.

### Standard Protocol Approvals, Registrations, and Patient Consents

Ethical approval was obtained by the Institutional Ethics Committees of Policlinic Agostino Gemelli at the Catholic University, Rome, Italy, where the surgery was performed. The protocol was also approved by the Italian Ministry of Health. Written informed consent and authorization for disclosure were signed by the patient. The clinical trial’s registration number on the online platform (www.ClinicalTrials.gov) is NCT02848846.

## Results

Both the invasive and non-invasive sensory feedback approaches were able to provide meaningful sensations to the patient (see Figures 2A,B). These sensations were then used by the subject during the experiments.

In order to assess the effect of adding sensory feedback to a hand prosthesis in a dual-task paradigm, we evaluated the memory span among the tested conditions. In particular, we found that the memory span was statistically lower (Span_baseline_ = 5.7 ± 0.6, Span_IF_ = 5.5 ± 0.6, Span_SF_ = 4.6 ± 1.3, Span_NF_ = 3.3 ± 0.7), when no sensory feedback was provided compared to baseline, IF and SF (Friedman test, *p* < 0.01; Figure 3A). On the contrary, when adding a sensory feedback, no change of the memory span with respect to baseline (Friedman test, *p* > 0.05; Figure 3A) was found. Looking at the functional performance estimation done by VET, firstly, the patient increased the percentage of unbroken and transferred blocks (manual accuracy, A) when using the sensory feedback (A_IF,C–OFF_ = 74.3 ± 4.6%, A_SF,C–OFF_ = 83.3 ± 12.7% and A_NF,C–OFF_ = 30.6 ± 27.4%), maintaining similar number of transferred blocks in total (gross manual dexterity, D) (D_IF,C–OFF_ = 15 ± 4, D_SF,C–OFF_ = 16 ± 2 and D_NF,C–OFF_ = 14.5 ± 3.5). Considering the gross manual dexterity, during the dual-task it was similar (D_IF,C–ON_ = 13.8 ± 2.2, D_SF,C–ON_ = 15.5 ± 1.5 and D_NF,C–ON_ = 14.3 ± 0.5 *p* > 0.05) to the one achieved during VET alone (without SDFT, C-OFF) in all conditions (Figure 3B, left). On the contrary, the patient’s manual accuracy was affected when a second task (with SDFT, C-ON) was added to VET. In particular, in NF condition the patient was totally unable to move unbroken blocks over the barrier showing a significant decrement of the manual accuracy (Friedman test, *p* < 0.01). When SF was provided in the dual-task paradigm, the performance significantly decreased (−17.3%, *p* < 0.05) showing that the cognitive effort was still high. Finally, in IF condition, the performance was not statistically different in the dual-task compared to the single task (Friedman test, *p* > 0.05), indicating that the neural sensory feedback was easier to integrate into the sensorimotor control. Furthermore, the response time (Figure 3C) in IF condition did not show any difference with the one measured at the baseline (Friedman test, *p* > 0.05 in all cases) unlike in SF and NF conditions.

**FIGURE 4:**
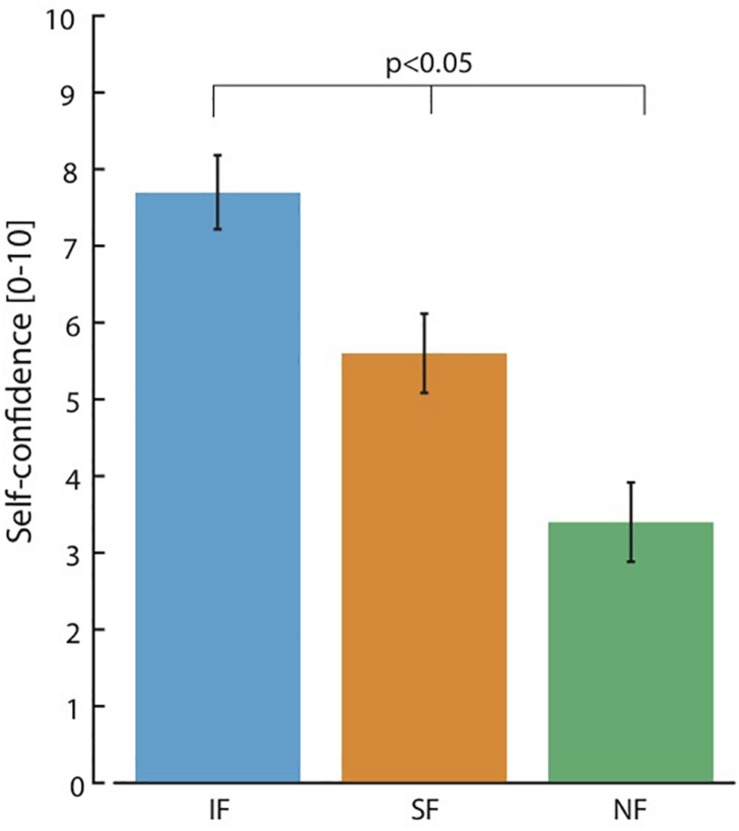
Self-confidence. A score of self-confidence was asked after the subject performed the task in each stimulation condition. All data are reported as mean values ± standard deviations. Friedman test, with Tukey-Kramer correction for multiple groups of data was performed. We performed 15 repetitions × 3 feedback conditions (Intraneural Feedback - IF, Superficial Feedback – SF and No Feedback – NF) × 2 cognitive conditions (with Cognitive task C-ON and without Cognitive task C-OFF).

Looking at the results regarding the self-confidence (Figure 4), during IF the score (7.7 ± 0.48) was significantly greater than in SF (5.6 ± 0.51) and in NF (3.4 ± 0.51) (Friedman test, *p* > 0.05). Confidence was also statistically higher when SF was provided compared to NF condition (Friedman test, *p* > 0.05).

## Discussion

Providing a tactile sensory feedback [superficial (SF) or intraneural (IF)] from sensors embedded in a hand prosthesis, while performing a motor task (VET), leads to maintain the memory span and count speed similar to the baseline (no motor task). It means that the short-term memory (STM) was overloaded by the dual-task in NF condition. The patient could not control the prosthesis, regulate the grip force relying only on visual feedback and recall the numbers simultaneously. Indeed, in NF condition also the performance in manual accuracy during the VET diminished significantly. Interestingly, the variability of the manual accuracy in NF condition was higher than in IF and SF. This was probably due to the poor reliability of the visual feedback to extract tactile information from the environment (e.g., grip force). Our results confirmed that the lack of a sensory inputs is reflected in increased cognitive effort (Williams et al., 2006; Raveh et al., 2018) during a dual-task. Looking at the sensory feedback type provided during the task, IF performed better than SF allowing to maintain high manual accuracy, with similar dexterity, even in the dual-task. Indeed, the manual accuracy in IF was statistically higher than SF only in C-ON condition. This evidence shows that the performance of an invasive or non-invasive technology is different when a daily activity (i.e., a task requiring more than one action simultaneously) is simulated. It could suggest that a more somatotopic, homologous and selective sensory feedback could be optimally exploited, and it could help more in daily living situations. Also, the response time in IF condition was on average lower than in NF and SF. This could indicate that the neural sensory feedback was easier to process, being more natural, showing a better integration into the sensorimotor control. The difference between IF and SF may be due to the different somatotopy of the sensation provided (*in loco* for SF and referred from the phantom hand in IF) and the sensation type (electricity for SF and vibration for IF). A somatotopic non-invasive stimulation (D’Anna et al., 2017; Osborn et al., 2018) should be tested and compared to verify these hypotheses. Further experiments are necessary to investigate these dependencies with more subjects and while performing different tasks also considering practice effects.

When a sensory feedback was provided, the subject had a higher self-confidence while performing the task. This result was consistent with previously presented experiments (Schiefer et al., 2018), in which transradial amputees were asked to give a score on their self-confidence while they were exploiting a bidirectional hand prosthesis in object identification tasks with and without the invasive sensory feedback. Interestingly also in our study, the subject was more confident when IF was provided respect to SF. This could be an ulterior evidence that a sensory feedback more somatotopic and more natural is better integrated and processed by the subject. In addition, the modal congruence of the stimulation-elicited percepts to the subject’s expectations may have influenced the relative weightings of the information.

Our results on a single patient need to be repeated in a large population of hand amputees in order to compare IF and SF techniques.

Moreover, recently, more biomimetic approaches were developed for restoring tactile sensory feedback (Valle et al., 2018), mimicking more closely the physiological behavior of natural sensors in the skin. Since in this work we used only the linear modulation of the current amplitude as a function of the prosthesis sensor readouts, it would be also interesting to investigate what happens with other encoding paradigms (Okorokova et al., 2018).

We believe that our findings support the hypothesis that intraneural stimulation providing sensory feedback to trans-radial amputee, could be effectively integrated into the sensorimotor control. Indeed, it improves the manual accuracy and dexterity of a hand prosthesis even when the motor control task is executed simultaneously with a cognitive task increasing the cognitive load.

## Data Availability Statement

The datasets generated for this study are available on request to the corresponding author.

## Ethics Statement

The studies involving human participants were reviewed and approved by Policlinico Gemelli and Italian Ministry of Research. The patients/participants provided their written informed consent to participate in this study. During the entire duration of our study, all experiments were conducted in accordance with EU guidelines and regulations.

## Author Contributions

GV, FC, MC, FP, and SM designed the experiment. GV, IS, and ED’A collected the data. GV analyzed the data, made the figures and drafted the manuscript. FC, MC, IS, FP, ED’A, TS, PR, and SM edited and revised the manuscript. GV, ED’A, and IS developed the software and the overall system integration. GG, RD, and PR selected the patient and were responsible for all the clinical aspects of the study. TS developed the TIME electrodes.

## Conflict of Interest

FP and SM hold shares of “SensArs Neuroprosthetics Sàrl,” a start-up company dealing with commercialization of neurocontrolled artificial limbs. MC and FC hold shares of “Prensilia,” a start-up company commercializing robotic hands and assessment tools. The remaining authors declare that the research was conducted in the absence of any commercial or financial relationships that could be construed as a potential conflict of interest.
